# Race and Ethnicity Representation in Phase 2/3 Oncology Clinical Trial Publications: A Systematic Review

**DOI:** 10.1001/jamahealthforum.2024.1388

**Published:** 2024-06-07

**Authors:** Kekoa Taparra, Ryan Benavente, Jonathan J. Shih, Megan Y. Gimmen, Paul Tominez, Kāʻeo Kekumano, Eric Pineda, Gisele Halualani, Henrietta Cakobau, Ethan B. Ludmir, Curtiland Deville, Jeffrey Peppercorn, Scarlett L. Gomez, Linda Bosserman, Fumiko Chino, Manali I. Patel, Chirag Shah

**Affiliations:** 1 Department of Radiation Oncology, Stanford Medicine, Stanford, California; 2 Harvard Medical School, Boston, Massachusetts; 3 School of Medicine, University of California, San Francisco; 4 Department of Human Developmental and Regenerative Biology, Harvard University, Cambridge, Massachusetts; 5 Tulane School of Medicine, New Orleans, Louisiana; 6 Department of Psychology, University of California, Berkeley; 7 Department of Cognitive Science, University of California, Berkeley; 8 Department of Gastrointestinal Radiation Oncology, MD Anderson Cancer Center, Houston, Texas; 9 Department of Radiation Oncology and Molecular Radiation Sciences, Johns Hopkins University, Baltimore, Maryland; 10 Massachusetts General Hospital, Boston, Massachusetts; 11 Helen Diller Family Comprehensive Cancer Center, University of California, San Francisco; 12 City of Hope Comprehensive Cancer Center, Duarte, California; 13 Department of Radiation Oncology, Memorial Sloan Kettering Cancer Center, New York, New York; 14 Department of Medicine, Stanford University School of Medicine, Stanford, California; 15 Medical Services, VA Palo Alto Health Care System, Palo Alto, California; 16 Department of Radiation Oncology, Cleveland Clinic, Cleveland, Ohio

## Abstract

**Importance:**

The five 1997 Office of Management and Budget races in the US include American Indian or Alaska Native, Asian, Black or African American, Native Hawaiian or Other Pacific Islander, and White, with Hispanic ethnicity. Despite the Affordable Care Act mandating Office of Management and Budget–based collecting and reporting standards, race and ethnicity publishing in medical journals is inconsistent, despite being necessary to achieve health equity.

**Objective:**

To quantify race and ethnicity reporting rates and calculate representation quotients (RQs) in published oncology clinical trials.

**Evidence Review:**

In this systematic review, PubMed and Embase were queried for phase 2/3 clinical trials of the 6 most common noncutaneous solid cancers, published between January 1, 2012, and December 31, 2022, in 4 high-impact journals. Trial characteristics were recorded. The RQs for each race and ethnicity were calculated by dividing the percent of representation in each clinical trial publication by the percent of year-matched, site-specific incident cancers in the US, compared with Kruskal-Wallis tests with Bonferroni correction (BC). Reporting was compared between journal publications and ClinicalTrials.gov.

**Findings:**

Among 1202 publications evaluated, 364 met inclusion criteria: 16 *JAMA*, 241 *Journal of Clinical Oncology*, 19 *Lancet*, and 88 *New England Journal of Medicine*. Publications included 268 209 patients (171 132 women [64%]), with a median of 356 (IQR, 131-800) patients per publication. Reported race and ethnicity included American Indian or Alaska Native in 52 (14%) publications, Asian in 196 (54%), Black or African American in 215 (59%), Hispanic in 67 (18%), Native Hawaiian or Other Pacific Islander in 28 (8%), and White in 254 (70%). Median RQ varied across race (*P* < .001 BC), with 1.04 (IQR, 0.09-4.77) for Asian, 0.98 (IQR, 0.86-1.06) for White, 0.42 (IQR, 0.12-0.75) for Black or African American, and 0.00 (IQR, 0.00-0.00) for both American Indian or Alaska Native and Native Hawaiian or Other Pacific Islander patients. Sensitivity analyses showed similar findings on subset analysis for US-only clinical trials. There was significantly less race and ethnicity reporting in the clinical trial publications compared with ClinicalTrials.gov documentation for American Indian or Alaska Native (14% vs 45%; *P* < .001 per McNemar χ^2^ test with continuity correction [MC]) and Native Hawaiian or Other Pacific Islander (8% vs 43%; *P* < .001 MC).

**Conclusions and Relevance:**

While most phase 2/3 oncology clinical trials published in high-impact journals report race and ethnicity, most did not report American Indian or Alaska Native and Native Hawaiian or Other Pacific Islander racial categories. Our findings support a call to action for consistent journal policies and transparent race and ethnicity reporting, in alignment with Affordable Care Act–concordant race and ethnicity federal reporting requirements.

## Key Points

**Question:**

What are the race and ethnicity reporting rates and representation quotients in published oncology clinical trials?

**Findings:**

In this systematic review of 364 phase 2/3 clinical trials of the 6 most common noncutaneous solid cancers, published between January 1, 2012, and December 31, 2022, in 4 high-impact journals, most did not report American Indian or Alaska Native and Native Hawaiian or Other Pacific Islander racial categories.

**Meaning:**

The findings of this study support a call to action for consistent journal policies and transparent race and ethnicity reporting, in alignment with Affordable Care Act–concordant race and ethnicity federal reporting requirements.

## Teaser

This systematic review assesses race and ethnicity reporting rates from 4 high-impact medical journals to examine patient representation by race and ethnicity compared with national cancer incidence.

## Introduction

Randomized clinical trial publications remain the standard for informing evidence-based treatment recommendations.^1^ However, oncology clinical trial enrollment has not reflected the diversity of the US population,^2^ even though patients from marginalized groups underrepresented in clinical trials accept invitations to participate in clinical trials if appropriately engaged.^3^ Ultimately, a lack of racial and ethnic diversity in published clinical trials challenges the generalizability of outcomes and can perpetuate health disparities by adversely affecting clinical practice guidelines.^4^

In 2010, the Affordable Care Act (ACA) Section 4302 required the US Department of Health and Human Services to establish race and ethnicity data collection and reporting standards, which adopted 5 federally defined race and ethnicity definitions per the 1997 Office of Management and Budget standards.^5^ To our knowledge, no study has evaluated oncology clinical trial representation across the 5 racial categories, specifically including American Indian or Alaska Native and Native Hawaiian or Other Pacific Islander populations. Therefore, the objectives of this study were to quantify race and ethnicity reporting rates from 4 high-impact medical journals and examine patient representation by race and ethnicity compared with national cancer incidence.

## Methods

In a systematic review, published phase 2/3 oncology clinical trials were evaluated in 4 high-impact journals (by Google Scholar/SCImago rankings): *JAMA*, *Journal of Clinical Oncology* (*JCO*), *Lancet*, and *New England Journal of Medicine* (*NEJM*), published between January 1, 2012, and December 31, 2022. The most common US noncutaneous solid cancers were included. Primary outcomes were race and ethnicity reporting rates and publication representativeness. Publications were excluded for not being phase 2/3 trials, having an international first author, evaluating multiple cancers, or not evaluating cancer treatment. Publications were assessed by 2 or more reviewers. This study followed the Preferred Reporting Items for Systematic Reviews and Meta-analyses (PRISMA) reporting guideline.

### Statistical Analysis

Representation quotients (RQs) were calculated by dividing the proportion of published patients by the proportion of year-matched, site-specific, US incident cancers. An RQ less than 1 signifies race or ethnicity underrepresented based on Cancer in North America data. Kruskal-Wallis tests with Bonferroni correction (BC) compared RQs. Multivariable logistic regression evaluated factors associated with race and ethnicity reporting, presented as adjusted odds ratios with 95% CIs. Journal publication race and ethnicity reporting and ClinicalTrials.gov federal entries were compared using the McNemar χ^2^ test with continuity correction (*P* MC). Analyses were performed using R, version 4.0.3 in RStudio, version 1.3.1093 (R Foundation for Statistical Computing).

## Results

### Publication Characteristics

Among 1202 publications identified using PubMed/Embase, 364 publications met inclusion criteria: 16 *JAMA*, 241 *JCO*, 19 *Lancet*, and 88 *NEJM* publications (Table 1; eFigure 1 in Supplement 1). The publications totaled 268 209 patients (171 132 women [64%]), with a median of 356 (IQR, 131-800) patients per publication. Most clinical trials in the publications were multicenter (95%), randomized (79%), evaluating systemic therapy (93%), industry-sponsored (76%), and phase 3 (60%).

**Table 1.** Characteristics of Phase 2/3 Oncology Clinical Trials Published in *JAMA*, *JCO*, *Lancet*, and *NEJM*

Characteristic	**No. (%)**				
	Overall (N = 364)	*JAMA* (n = 16)	*JCO* (n = 241)	*Lancet* (n = 19)	*NEJM* (n = 88)
Any race and ethnicity reporting	260 (71)	13 (81)	173 (72)	15 (79)	59 (67)
Race and ethnicity reporting					
American Indian or Alaska Native	52 (14)	6 (38)	37 (15)	2 (11)	7 (8)
Asian	196 (54)	11 (69)	122 (51)	12 (63)	51 (58)
Black	215 (59)	12 (75)	143 (59)	13 (68)	47 (53)
Hispanic	67 (18)	5 (31)	54 (22)	4 (21)	4 (5)
Native Hawaiian or Other Pacific Islander	28 (8)	4 (25)	19 (8)	2 (11)	3 (3)
White	254 (70)	13 (81)	172 (71)	15 (79)	54 (61)
Multiracial	22 (6)	2 (12)	14 (6)	4 (21)	2 (2)
Other	165 (45)	5 (31)	110 (46)	10 (53)	40 (45)
Missing	145 (40)	9 (56)	89 (37)	12 (63)	35 (40)
Patients					
Total patients, No.	268 209	11 139	145 838	18 908	92 324
Per publication, median (IQR)	356 (131-800)	450 (217-740)	236 (90-658)	850 (302-1253)	688 (399-890)
Men					
Total patients, No.	97 077 (36)	5551 (50)	50 838 (35)	9783 (52)	30 906 (33)
Per publication, median (IQR)	70 (0-389)	214 (27-369)	44 (0-210)	428 (127-752)	196 (1-560)
Women					
Total patients, No.	171 132 (64)	5588 (50)	95 000 (65)	9125 (48)	61 419 (67)
Per publication, median (IQR)	118 (20-323)	187 (42-519)	80 (15-236)	246 (75-409)	213 (65-622)
Year					
2012-2017	185 (51)	11 (69)	128 (53)	11 (58)	35 (40)
2018-2022	179 (49)	5 (31)	113 (47)	8 (42)	53 (60)
Trial phase					
2	146 (40)	4 (25)	124 (51)	2 (11)	16 (18)
3	218 (60)	12 (75)	117 (49)	17 (89)	72 (82)
US region					
Northeast	158 (43)	6 (38)	100 (41)	6 (32)	46 (52)
Midwest	47 (13)	3 (19)	33 (14)	2 (11)	9 (10)
South	111 (30)	2 (12)	81 (34)	4 (21)	24 (27)
West	48 (13)	5 (31)	27 (11)	7 (37)	9 (10)
Cancer site					
Breast	116 (32)	4 (25)	78 (32)	4 (21)	30 (34)
Colorectal	20 (6)	6 (38)	11 (5)	0	3 (3)
Endometrial	14 (3.8)	0	12 (5.0)	0	2 (2.3)
Kidney/bladder	60 (16)	1 (6.2)	39 (16)	6 (32)	14 (16)
Lung	88 (24)	1 (6.2)	57 (24)	6 (32)	24 (27)
Prostate	66 (18)	4 (25)	44 (18)	3 (16)	15 (17)
Primary end point					
Survival	208 (61)	7 (44)	120 (54)	14 (78)	67 (80)
Treatment response	68 (20)	1 (6)	51 (23)	2 (11)	14 (17)
Trial design^a^					
Only US participants	172 (47)	9 (56)	148 (61)	3 (16)	12 (14)
Multicenter	345 (95)	14 (88)	225 (93)	18 (95)	88 (100)
Randomized	289 (79)	15 (94)	176 (73)	19 (100)	79 (90)
Blinded	112 (31)	10 (62)	60 (25)	9 (47)	33 (38)
Industry sponsored	276 (76)	7 (44)	177 (73)	15 (79)	77 (88)
Systemic therapy	337 (93)	11 (69)	226 (94)	15 (79)	85 (97)
Radiotherapy	29 (8)	2 (12)	22 (9)	3 (16)	2 (2)
Surgery	12 (3)	2 (12)	5 (2)	1 (5)	4 (5)

Abbreviations: *JCO*, *Journal of Clinical Oncology*; *NEJM*, *New England Journal of Medicine*.

^a^ The trial design variables are independently recorded as binary yes or no (not mutually exclusive).

### Race and Ethnicity Reporting

Table 1 and the Figure show the race and ethnicity reporting overall and stratified by journal. Reporting of any race was found in 260 publications (71%): 13 (81%) *JAMA*, 15 (79%) *Lancet*, 173 (72%) *JCO*, and 59 (67%) *NEJM*. The reporting of all 5 ACA-concordant race categories varied widely across the oncology clinical trial publications, with race and ethnicity reporting generally highest in *JAMA* and lowest in *NEJM*. Reporting varied by race and ethnicity from 28 (8%) for Other Pacific Islander populations to 254 (70%) for the White populations.

**Figure.**
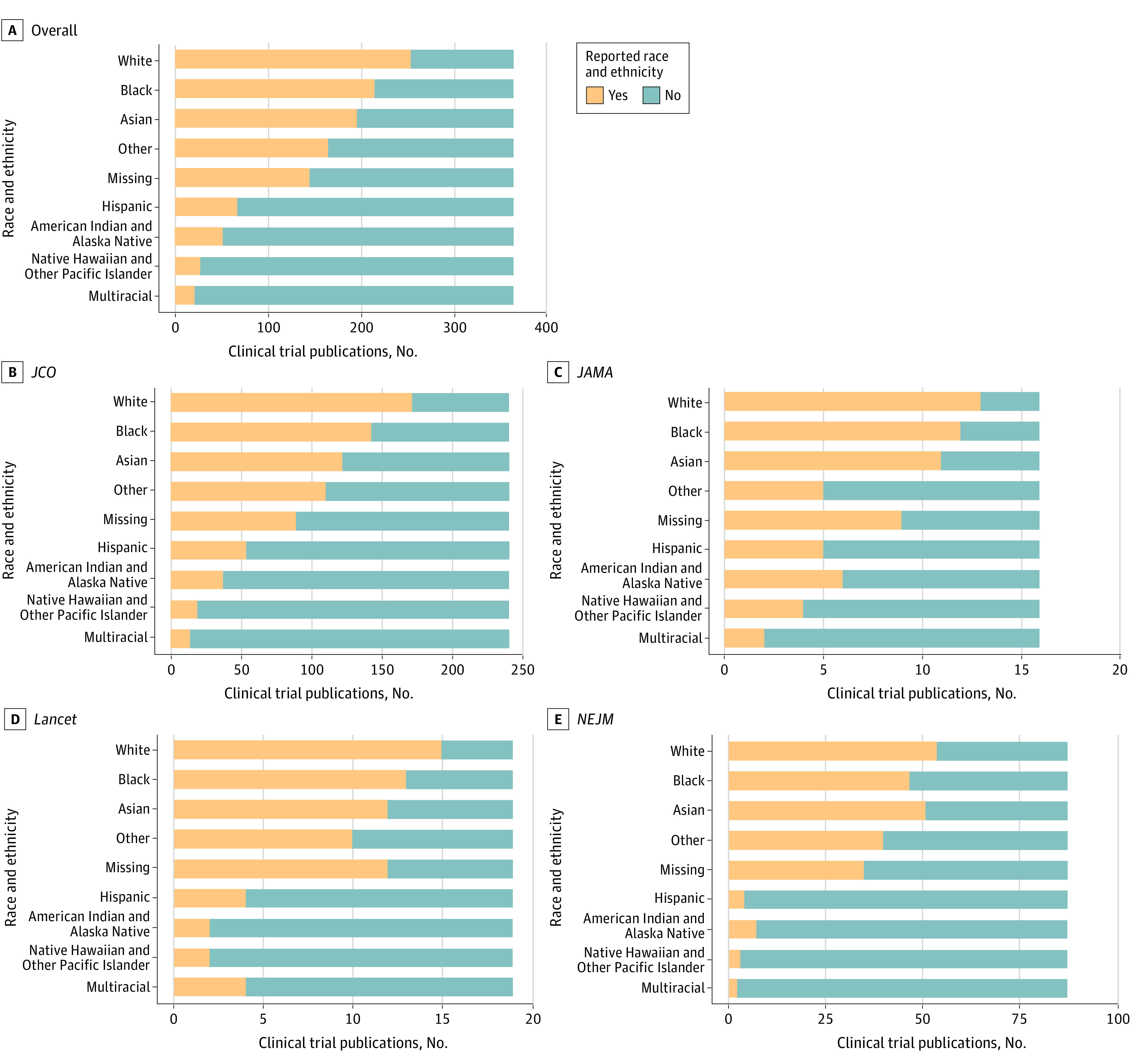
Proportion of Phase 2/3 Cancer Clinical Trial Publications That Report Race and Ethnicity Published in the *Journal of Clinical Oncology* (*JCO*), *JAMA*, *Lancet*, and the *New England Journal of Medicine* (*NEJM*) Between 2012 and 2022

### Representation Quotients

Table 2 and eFigure 2 in Supplement 1 display the median RQ of phase 2/3 US oncology clinical trial publications, which was lowest across all cancers among both American Indian or Alaska Native and Native Hawaiian or Other Pacific Islander patients at 0.00 (IQR, 0.00-0.00) and highest among Asian patients at 1.04 (IQR, 0.09-4.77; *P* < .001 BC). Median RQ for Hispanic patients was 0.60 (IQR, 0.37-0.82), which was lower than non-Hispanic patients at 1.04 (IQR, 1.01-1.06; *P* < .001 BC). Across race, all cancer sites had significantly different RQs (*P* < .001 BC) except for endometrial cancer. For Hispanic ethnicity, RQs were significantly different for breast, colorectal, and prostate (*P* < .05 BC) cancers. Multivariable regression found US-based trials were most likely to publish race and ethnicity (adjusted odds ratio, 1.67; 95% CI, 1.01-2.80) (eTable 1 in Supplement 1). Disparities in race and ethnicity reporting persisted within the US-only subset sensitivity analysis. American Indian or Alaska Native and Native Hawaiian or Other Pacific Islander race was reported less in journal publications compared with ClinicalTrials.gov (*P* < .001 MC) (eTable 2 in Supplement 1).

**Table 2.** Representation Quotients of Published Phase 2/3 Cancer Clinical Trials by Cancer Site and Race and Ethnicity^a^

Cancer	Representation quotient, median (IQR)												
	Race									Ethnicity			
	American Indian or Alaska Native	Asian	Black	Native Hawaiian or Other Pacific Islander	White	Other	Missing	*P* value	*P* value, BC	Hispanic	Non-Hispanic	*P* value	*P* value, BC
Breast	0.00 (0.00-0.00)	0.83 (0.12-2.32)	0.51 (0.12-0.80)	0.00 (0.00-0.00)	0.97 (0.83-1.04)	3.09 (0.00-9.77)	0.31 (0.00-5.37)	<.001	<.001	0.62 (0.40-0.91)	1.04 (1.01-1.06)	.002	.01
Colorectal	0.00 (0.00-0.00)	0.93 (0.05-1.64)	0.46 (0.11-0.87)	0.00 (0.00-0.00)	1.03 (0.97-1.09)	0.00 (0.00-1.58)	1.28 (0.00-4.53)	<.001	<.001	0.60 (0.46-0.67)	1.05 (1.04-1.06)	<.001	.004
Endometrial	0.67 (0.00-1.88)	1.03 (0.63-1.98)	0.75 (0.50-0.95)	0.00 (0.00-1.23)	0.96 (0.91-1.02)	0.39 (0.00-4.71)	2.25 (0.00-3.91)	.40	>.99	0.61 (0.58-0.66)	1.05 (1.05-1.06)	.008	.06
Kidney/bladder	0.00 (0.00-0.00)	1.10 (0.15-3.95)	0.35 (0.01-0.65)	0.00 (0.00-0.00)	1.00 (0.90-1.05)	1.29 (0.00-6.11)	1.96 (0.00-5.60)	<.001	<.001	0.57 (0.36-0.82)	1.03 (1.02-1.06)	.01	.09
Lung	0.00 (0.00-0.00)	5.19 (0.93-7.87)	0.25 (0.08-0.61)	0.00 (0.00-0.00)	0.92 (0.84-0.99)	6.76 (0.45-13.72)	0.34 (0.00-6.62)	<.001	<.001	0.82 (0.37-1.65)	1.01 (0.97-1.03)	.44	>.99
Prostate	0.00 (0.00-0.00)	0.45 (0.00-1.25)	0.54 (0.24-0.76)	0.00 (0.00-0.00)	1.07 (0.99-1.12)	0.51 (0.00-1.30)	2.02 (0.00-5.26)	<.001	<.001	0.41 (0.28-0.61)	1.05 (1.04-1.07)	<.001	<.001
Overall	0.00 (0.00-0.00)	1.04 (0.09-4.77)	0.42 (0.12-0.75)	0.00 (0.00-0.00)	0.98 (0.86-1.06)	1.26 (0.00-8.25)	0.65 (0.00-5.37)	<.001	<.001	0.60 (0.37-0.82)	1.04 (1.01-1.06)	<.001	<.001

Abbreviation: BC, Bonferroni correction.

^a^ Representation quotients are calculated from the proportion of patients by race and ethnicity enrolled in a published clinical trial divided by the reported United States North American Association of Central Cancer Registries (NAACCR) Cancer in North America year-matched site-specific cancer incidence by race. A representation quotient of 0 signifies race or ethnicity without any published representation relative to Cancer in North America data. Representation quotients of 1 represent equal representation in a clinical trial compared with expected US NAACCR year-matched site-specific cancer incidence. *P* values were calculated using the Kruskal-Wallis rank-sum test.

## Discussion

To our knowledge, we conducted the first analysis of oncology clinical trial representation according to federal standards, including American Indian or Alaska Native and Native Hawaiian or Other Pacific Islander patients. Among the 5 racial and ethnic categories, Asian and White participants were most frequently reported, while American Indian or Alaska Native and Native Hawaiian or Other Pacific Islander patients were least reported. Moreover, American Indian or Alaska Native and Native Hawaiian or Other Pacific Islander representativeness was not merely low, but 0 among published oncology clinical trials assessed. Our findings support a call-to-action for medical researchers to uphold policies that adhere to ACA-concordant reporting requirements to prevent erasure of Indigenous health disparities.^6^

American Indian or Alaska Native and Native Hawaiian or Other Pacific Islander patients are known to have the lowest life expectancy in the US and face underrecognized health disparities.^6^ These populations lack access to clinical trials given their isolated geographic residence across Tribal Nations and Pacific regions.^7,8,9^ American Indian or Alaska Native patients living on reservations face barriers to clinical trials, including long travel times, lodging expenses, and conflicts between Western medicine and traditional healing knowledge.^9^ Native Hawaiian or Other Pacific Islander patients in the Pacific live thousands of miles away from cancer facilities.

Adoption of patient health navigators and community health workers has great potential to help facilitate coordinated continuity of care for patients with cancer who are eligible for clinical trials. American Indian or Alaska Native and Native Hawaiian or Other Pacific Islander data omission is a form of structural racism that perpetuates Indigenous health disparities.^8,10^ By adhering to ACA-concordant race and ethnicity reporting requirements, medical journals have the opportunity to promote transparent, consistent, and accurate data reporting for the most marginalized populations. With limited attention to Indigenous inclusion and representation in clinical trials, there is a clear need for funding and innovative strategies to improve Indigenous clinical trial enrollment.

Our analysis echoes more than 3 decades of previous studies that show that Black or African American and Hispanic patients remain consistently underrepresented in cancer clinical trials, persisting even after US Food and Drug Administration approval.^2,11,12^ Clinician-imposed selection bias is also known to contribute to disproportionate race and ethnicity representation in clinical trials.^13^ Broadening selection criteria to include a wider range of prognosis or comorbidity burden may also increase clinical trial enrollment across diverse populations.^14,15^

### Strengths and Limitations

Strengths of this study include the inclusion of all 5 ACA-concordant race and ethnic reporting categories, the comprehensive nature of the RQ analysis across 6 major cancers, and the use of the Cancer in North America dataset for standardization of national cancer incidence. This study has limitations. First, a major limitation is that, while inclusive of more than 97% of the US population, large cancer databases, including the Cancer in North America database, substantially underestimate American Indian and Alaska Native cancer incidence given the lack of integration within the Indian Health Service or cross-validation with Tribal systems; nonetheless, the 0.00 American Indian and Alaska Native RQ would likely persist. Second, we used US cancer incidence, which may have skewed international trials, which was the rationale for our sensitivity analyses suggesting a consistent finding among the US-only studies. Third, we focused on 4 journals and acknowledge the inclusion of other journals may improve generalizability. Improving race and ethnicity reporting at these high-impact journals could set new standards and motivate trialists to improve their race and ethnicity collection and reporting practices.

## Conclusions

The findings of this systematic review suggest that, when included per federal guidelines, American Indian or Alaska Native and Native Hawaiian or Other Pacific Islander patients are the least reported in cancer clinical trial publications. Developing rigorous publication standards by journal editors may help improve equitable representation in US oncology clinical trials. Bolstering awareness of US federal race and ethnicity standards ensures patients from all racial and ethnic backgrounds can be equitably included moving forward.

## Notes

Supplement 1. **eTable 1.** Univariable and Multivariable Logistic Regression for Significant Predictors of Race and Ethnicity Reporting **eTable 2.** Comparison Between Indigenous Race Reporting Between Clinical Trial Publication and ClinicalTrials.org **eFigure 1.** PRISMA Flow Diagram of Included and Excluded Oncology Clinical Trial Publications **eFigure 2.** Violin Plot of Yearly Composite Representation Quotients of Published Phase 2/3 Oncology Clinical Trials by Cancer Site and the 5 Federally Recognized Race Categories

Supplement 2.
Data Sharing Statement

## References

[1] Oyer RA, Hurley P, Boehmer L, . Increasing racial and ethnic diversity in cancer clinical trials: an American Society of Clinical Oncology and Association of Community Cancer Centers joint research statement. J Clin Oncol. 2022;40(19):2163-2171. doi:10.1200/JCO.22.00754 35588469

[2] Duma N, Vera Aguilera J, Paludo J, . Representation of minorities and women in oncology clinical trials: review of the past 14 years. J Oncol Pract. 2018;14(1):e1-e10. doi:10.1200/JOP.2017.025288 29099678

[3] Unger JM, Hershman DL, Till C, . “When offered to participate”: a systematic review and meta-analysis of patient agreement to participate in cancer clinical trials. J Natl Cancer Inst. 2021;113(3):244-257. doi:10.1093/jnci/djaa155 33022716 PMC7936064

[4] Gomez SL, Tsai CJ. Is representation enough or should we be targeting equitable inclusion? Nat Rev Clin Oncol. 2022;19(7):429-430. doi:10.1038/s41571-022-00635-z 35411091

[5] United States Department of Health and Human Services. HHS implementation guidance on data collection standards for race, ethnicity, sex, primary language, and disability status. ASPE. October 30, 2011. Accessed December 10, 2023. https://aspe.hhs.gov/reports/hhs-implementation-guidance-data-collection-standards-race-ethnicity-sex-primary-language-disability-0

[6] Taparra K, Pellegrin K. Data aggregation hides Pacific Islander health disparities. Lancet. 2022;400(10345):2-3. doi:10.1016/S0140-6736(22)01100-X 35717993

[7] Pineda E, Benavente R, Gimmen MY, DeVille NV, Taparra K. Cancer disparities among Pacific Islanders: a review of sociocultural determinants of health in the Micronesian region. Cancers (Basel). 2023;15(5):1392. doi:10.3390/cancers15051392 36900185 PMC10000177

[8] Taparra K, Miller RC, Deville C Jr. Navigating Native Hawaiian and Pacific Islander cancer disparities from a cultural and historical perspective. JCO Oncol Pract. 2021;17(3):130-134. doi:10.1200/OP.20.00831 33497251

[9] Melkonian SC, Jim MA, Pete D, . Cancer disparities among non-Hispanic urban American Indian and Alaska Native populations in the United States, 1999-2017. Cancer. 2022;128(8):1626-1636. doi:10.1002/cncr.34122 35119703 PMC10929659

[10] Morey BN, Chang RC, Thomas KB, . No equity without data equity: data reporting gaps for Native Hawaiians and Pacific Islanders as structural racism. J Health Polit Policy Law. 2022;47(2):159-200. doi:10.1215/03616878-9517177 34522960 PMC10959240

[11] Deville C Jr, Borno HT. Declining representation and reporting of racial and ethnic minority patients in prostate cancer clinical trials despite persistent health disparities—where progress confronts limitations. JAMA Oncol. 2023;9(2):175-177. doi:10.1001/jamaoncol.2022.6749 36520457

[12] Varma T, Wallach JD, Miller JE, . Reporting of study participant demographic characteristics and demographic representation in premarketing and postmarketing studies of novel cancer therapeutics. JAMA Netw Open. 2021;4(4):e217063. doi:10.1001/jamanetworkopen.2021.7063 33877309 PMC8058642

[13] Joseph G, Dohan D. Diversity of participants in clinical trials in an academic medical center: the role of the “Good Study Patient?” Cancer. 2009;115(3):608-615. doi:10.1002/cncr.24028 19127544

[14] Taparra K, Qu V, Pollom E. Disparities in survival and comorbidity burden between Asian and Native Hawaiian and Other Pacific Islander patients with cancer. JAMA Netw Open. 2022;5(8):e2226327. doi:10.1001/jamanetworkopen.2022.26327 35960520 PMC9375163

[15] Akimoto K, Taparra K, Brown T, Patel MI. Diversity in cancer care: current challenges and potential solutions to achieving equity in clinical trial participation. Cancer J. 2023;29(6):310-315. doi:10.1097/PPO.0000000000000675 37963364

