# Errors in a Table and Figure

**DOI:** 10.1001/jamahealthforum.2024.2301

**Published:** 2024-07-12

The Original Investigation “Race and Ethnicity Representation in Phase 2/3 Oncology Clinical Trial Publications: A Systematic Review,”^1^ published June 7, 2024, included errors in the Figure and Table 1. In each panel of the Figure, the labels for Hispanic and Native American and Alaska Native representation in oncology clinical trials were transposed. In Table 1, under Race and Ethnicity, the row indicating representation of Hispanic participants was missing and has been added. This article has been corrected.^1^
