# Supplementary material for: Race and Ethnicity Representation in Phase 2/3 Oncology Clinical Trial Publications: A Systematic Review
Source: JAMA Health Forum. 2024 Jun 7;5(6):e241388. doi: 10.1001/jamahealthforum.2024.1388 (PMC11161837; doi:10.1001/jamahealthforum.2024.1388)
Supplement: Supplement 1. — eTable 1. Univariable and Multivariable Logistic Regression for Significant Predictors of Race and Ethnicity Reporting eTable 2. Comparison Between Indigenous Race Reporting Between Clinical Trial Publication and ClinicalTrials.org eFigure 1. PRISMA Flow Diagram of Included and Excluded Oncology Clinical Trial Publications eFigure 2. Violin Plot of Yearly Composite Representation Quotients of Published Phase 2/3 Oncology Clinical Trials by Cancer Site and the 5 Federally Recognized Race Categories [file jamahealthforum-e241388-s001.pdf]

Supplementary Online Content

Taparra K, Benavente R, Shih JJ, et al. Race and ethnicity representation in phase 2/3 oncology clinical trial publications in the US. *JAMA Health Forum*. 2024;5(6):e241388. doi:10.1001/jamahealthforum.2024.1388

eTable 1. Univariable and Multivariable Logistic Regression for Significant Predictors of Race and Ethnicity Reporting

eTable 2. Comparison Between Indigenous Race Reporting Between Clinical Trial Publication and ClinicalTrials.org

eFigure 1. PRISMA Flow Diagram of Included and Excluded Oncology Clinical Trial Publications

eFigure 2. Violin Plot of Yearly Composite Representation Quotients of Published Phase 2/3 Oncology Clinical Trials by Cancer Site and the 5 Federally Recognized Race Categories

This supplementary material has been provided by the authors to give readers additional information about their work.

© 2024 Taparra K et al. *JAMA Health Forum.*

Characteristic	(vs Reference Group)	Unadjusted LR			Adjusted LR		
		OR	95% CI	P	aOR	95% CI	P
**Any Race/Ethnicity[1]**							
Only US Participants	(vs International)	1.40	(0.88-2.22)	0.153	1.67	(1.01-2.80)	0.048
**White[2]**							
Kidney/Bladder	(vs Breast Cancer)	0.56	(0.29-1.07)	0.089	0.49	(0.25-0.96)	0.039
Only US Participants	(vs International)	1.52	(0.97-2.41)	0.067	1.78	(1.02-3.13)	0.045
Multicenter Trial	(vs Single Center)	2.17	(0.84-5.55)	0.107	3.62	(1.31-9.99)	0.012
**Black[3]**							
Only US Participants	(vs International)	1.40	(0.92-2.14)	0.113	1.75	(1.08-2.87)	0.023
Multicenter Trial	(vs Single Center)	2.06	(0.81-5.45)	0.126	3.34	(1.24-9.45)	0.018
**AI/AN[4]**							
NEJM	(vs JAMA)	0.14	(0.04-0.52)	0.095	0.21	(0.05-0.94)	0.040
Endometrial	(vs Breast Cancer)	5.94	(1.72-20.0)	0.053	11.3	(2.70-48.6)	<0.001
Only US Participants	(vs International)	2.38	(1.30-4.48)	0.005	3.25	(1.52-7.23)	0.003
**NHPI[5]**							
West	(vs Northeast)	4.93	(1.83-13.8)	<0.001	3.78	(1.28-11.6)	0.017
Endometrial	(vs Breast Cancer)	6.23	(1.44-24.7)	0.117	10.3	(1.82-58.9)	0.008
**Hispanic[6]**							
NEJM	(vs JAMA)	0.10	(0.02-0.45)	<0.001	0.14	(0.03-0.67)	0.014
2018-2022	(vs 2012-2017)	1.96	(1.15-3.42)	0.014	2.36	(1.31-4.31)	0.005
Industry Sponsored	(vs Not Sponsored)	0.35	(0.20-0.62)	<0.001	0.50	(0.27-0.95)	0.033

Covariates used in multivariable model after forward stepwise selection

1. Adjusted for US region, cancer site, multicenter trial, having only US patients, and blinding.
2. Adjusted for journal, cancer site, multicenter trial, and having only US patients.
3. Adjusted for cancer site, multicenter trial, having only US patients, randomization, blinding, and industry sponsorship.
4. Adjusted for journal, US region, cancer site, multicenter trial, having only US patients, randomization, and industry sponsorship.
5. Adjusted for journal, US region, trial phase, cancer site, randomization, blinding, and industry sponsorship.
6. Adjusted for journal, year, having only US patients, industry sponsorship, and treatment response primary endpoint.

eTable 1. Univariable and multivariable logistic regression for significant predictors of race and ethnicity reporting. Covariables included journal, US region, year, trial phase, cancer site, only US participation, multicenter trial, randomization, blinding, and industry sponsorship, survival endpoint primary endpoint, and treatment response primary endpoint. The full list of covariates meeting this threshold is reported numerically in the footnote by race. Asian race reporting did not have any statistically significant covariates on multivariable logistic regression. Abbreviations: aOR=Adjusted Odds Ratio; 95%CI=95% Confidence Interval; AI/AN=American Indian and Alaska Native; NHPI=Native Hawaiian and Other Pacific Islander; NEJM=New England Journal of Medicine; JAMA=Journal of the American Medical Association.

© 2024 Taparra K et al. *JAMA Health Forum.*

Race Reported	Publication Reported	ClinicalTrials.gov Reported	P_{MC}
**AI/AN**			<0.001
No	312 (86%)	199 (55%)	
Yes	52 (14%)	165 (45%)	
**NHPI**			<0.001
No	336 (92%)	208 (57%)	
Yes	28 (7.7%)	156 (43%)	

eTable 2. Comparison between Indigenous Race reporting between clinical trial publication and ClinicalTrials.org. AI/AN and NHPI were chosen for evaluation as the only two groups having the lowest RQ values, with an overall median RQ of 0.00. Statistics reported as n (%). McNemar's Chi-squared test with continuity correction was used to calculate the *P*-value (P_{MC}). Abbreviations: AI/AN=American Indian and Alaska Native; NHPI=Native Hawaiian and other Pacific Islander.

© 2024 Taparra K et al. *JAMA Health Forum.*

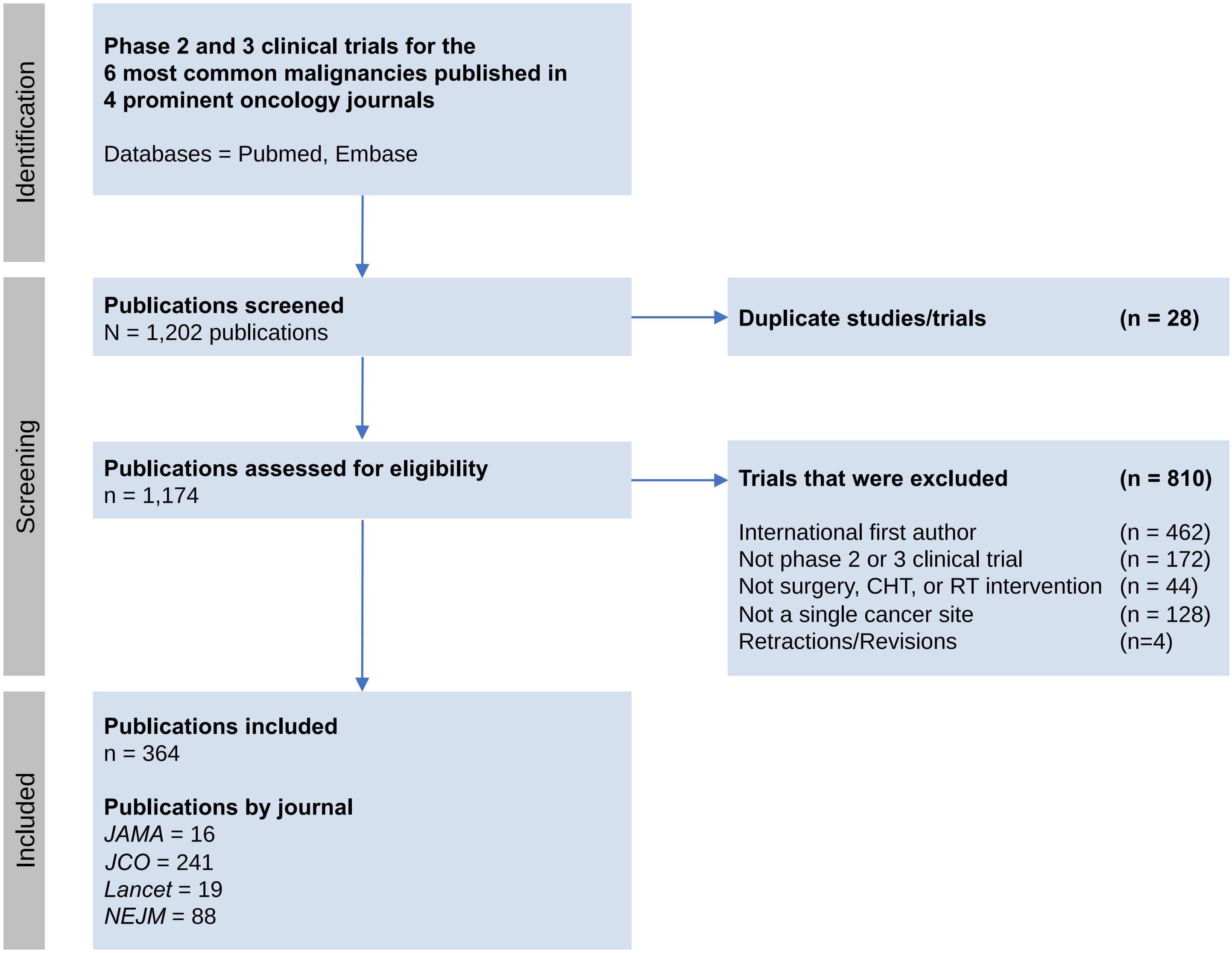

eFigure 1. PRISMA Flow diagram of included and excluded oncology clinical trial publications. Initial publication identification was obtained using Pubmed and Embase searches for phase 2/3 clinical trials of the most common non-cutaneous solid malignancies in the United States published in *JAMA*, *JCO*, *Lancet*, or *NEJM*, between 2012-2022. Cancers included were lung, breast, prostate, endometrial, kidney/bladder, and colorectal cancers. All papers were reviewed by 2 independent reviewers, discrepancies were evaluated and decided by KT. Abbreviations: JAMA=Journal of the American Medical Association; JCO=Journal of Clinical Oncology; NEJM=New England Journal of Medicine; CHT=Chemotherapy; RT=Radiation Therapy.

© 2024 Taparra K et al. *JAMA Health Forum.*

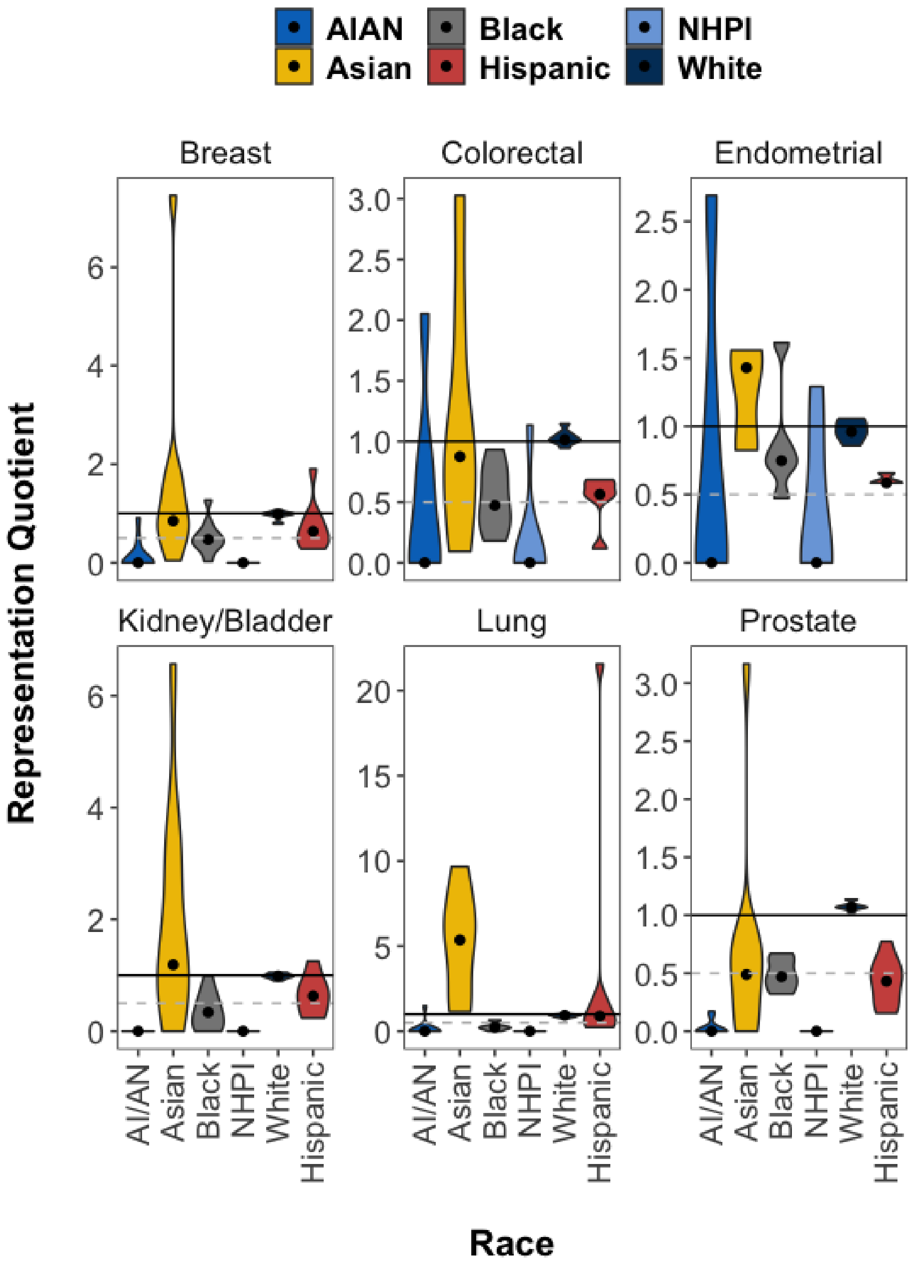

eFigure 2. Violin plot of yearly composite representation quotients of published phase 2/3 oncology clinical trials by cancer site and the 5 federally recognized race categories. Yearly composite RQ values represent the proportion of patients included in clinical trials by race compared to year-matched site-specific NAACCR CiNA data. Black circles represent median yearly composite representation quotients by race, 2012-2022. Abbreviations: AI/AN=American Indian and Alaska Native; NHPI=Native Hawaiian and other Pacific Islanders; NAACCR=North American Association of Central Cancer Registries; CINA=Cancer in North America.

© 2024 Taparra K et al. *JAMA Health Forum.*
