# Supplementary material for: Race and Ethnicity Representation in Phase 2/3 Oncology Clinical Trial Publications: A Systematic Review
Source: JAMA Health Forum. 2024 Jun 7;5(6):e241388. doi: 10.1001/jamahealthforum.2024.1388 (PMC11161837; doi:10.1001/jamahealthforum.2024.1388)
Supplement: Supplement 2. — Data Sharing Statement [file jamahealthforum-e241388-s002.pdf]

Data Sharing Statement

Taparra. Race and Ethnicity Representation in Phase 2/3 Oncology Clinical Trial Publications. *JAMA Health Forum*. Published June 07, 2024. doi:10.1001/jamahealthforum.2024.1388

Data
Data available: No

Additional Information
Explanation for why data not available: Papers included in the study are publicly available.
